# Two rare cases of synchronous and metachronous colonic metastases in patients with advanced gastric cancer

**DOI:** 10.1186/s12957-018-1323-8

**Published:** 2018-01-31

**Authors:** Wei-Chih Su, Hsiang-Lin Tsai, Chun-Chieh Wu, Shan-Yin Tsai, Yung-Sung Yeh, Cheng-Jen Ma, Jaw-Yuan Wang

**Affiliations:** 1 Division of Colorectal Surgery, Department of Surgery, Kaohsiung Medical University Hospital, No. 100, Tzyou 1st Road, Kaohsiung, 807 Taiwan; 2 Graduate Institute of Clinical Medicine, College of Medicine, Kaohsiung Medical University, Kaohsiung, Taiwan; 3 Department of Surgery, Faculty of Medicine, College of Medicine, Kaohsiung Medical University, Kaohsiung, Taiwan; 4 Department of Pathology, Kaohsiung Medical University Hospital, Kaohsiung, Taiwan; 5 Division of Trauma and Surgical Critical Care, Department of Surgery, Kaohsiung Medical University Hospital, Kaohsiung Medical University, Kaohsiung, Taiwan; 6 Division of General and Digestive Surgery, Department of Surgery, Kaohsiung Medical University Hospital, Kaohsiung Medical University, Kaohsiung, Taiwan; 7 Graduate Institute of Medicine, College of Medicine, Kaohsiung Medical University, Kaohsiung, Taiwan; 8 Center for Biomarkers and Biotech Drugs, Kaohsiung Medical University, Kaohsiung, Taiwan; 9 Research Center for Environmental Medicine, Kaohsiung Medical University, Kaohsiung, Taiwan; 10 Research Center for Natural Products & Drug Development, Kaohsiung Medical University, Kaohsiung, Taiwan

**Keywords:** Gastric cancer, Synchronous colonic metastasis, Metachronous colonic metastasis

## Abstract

**Background:**

Patients with advanced gastric cancer (GC) may ultimately die because GC mostly leads to synchronous or metachronous metastasis. However, colonic metastasis of GC is extremely rare. According to a PubMed search of papers published from May 1968 to March 2017, only 21 patients with GC (10 patients from 10 case reports and 11 patients from a retrospective study) have been found to have colonic metastasis. In this report, we present two cases of synchronous and metachronous colonic metastases of advanced GC.

**Case presentation:**

Two patients with advanced GC received a diagnosis of colonic metastasis based on colonoscopic findings and computed tomography images, and the diagnosis was confirmed through pathological immunohistochemical analysis. Herein, we describe the management and outcomes of these metastases.

**Conclusions:**

Submucosal swelling and segmental bowel wall thickening observed through colonoscopy in patients with advanced GC might indicate colonic metastasis.

## Background

Gastric carcinoma is the forth leading cause of cancer-related deaths worldwide [1]. In the advanced stages of this disease, patients may develop either synchronous or metachronous metastasis. The most common sites of gastric cancer (GC) metastasis are the liver, peritoneum, lungs, and bones [2]. According to our review of the literature published between May 1968 and March 2017, 21 patients with GC (10 patients from 10 case reports and 11 patients from a retrospective study) received a diagnosis of colonic metastasis [3–13]. In this report, we present two rare cases of synchronous and metachronous colonic metastases of advanced GC and report their diagnoses, management, and clinical outcomes.

## Case presentation

### Case 1: synchronous colonic metastasis of advanced GC

**Fig. 1:**
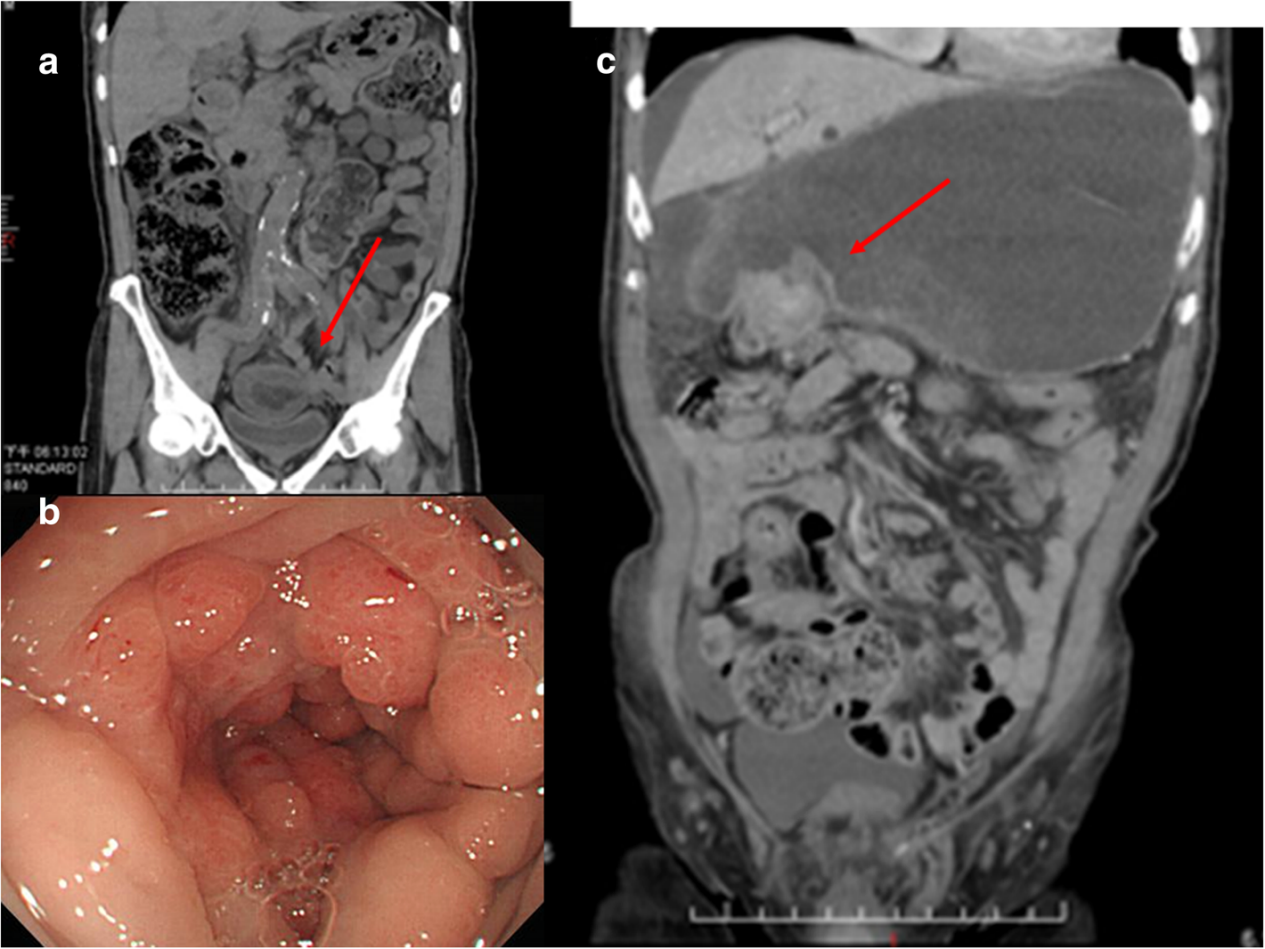
Synchronous colonic metastasis of advanced GC. **a** Abdominal CT showed suspected rectosigmoid colon cancer with partial obstruction; **b** colonoscopy revealed rectosigmoid colon wall thickening and partially obstructed mucosal swelling; **c** abdominal CT displayed circumferential thickening of the pylorus and marked stomach distension, indicating the possibility of GC

**Fig. 2:**
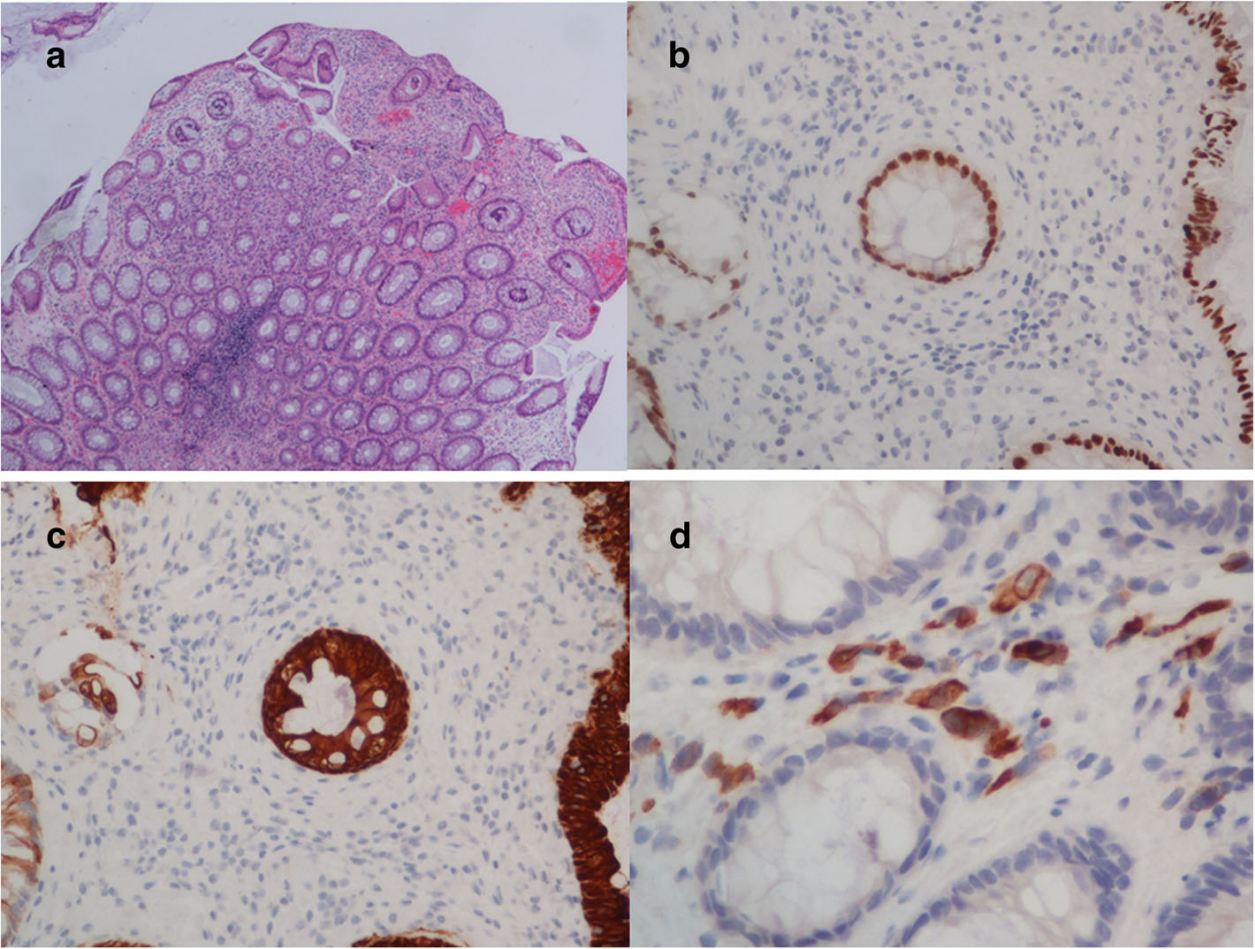
**a** Poorly differentiated carcinoma involving colonic submucosa hematoxylin and eosin staining (HE) (× 40); **b** CDX-2 staining with negative results (× 100); **c** CK20 staining with negative results (× 100); and **d** CK7 staining with positive results (× 400)

A 77-year-old man visited our emergency department with acute abdominal pain for 2 days. After initial evaluation, abdominal computed tomography (CT) was performed, which revealed diffuse wall edema of the rectosigmoid colon (Fig. 1a). Rectosigmoid colon cancer with partial obstruction was suspected, and transverse colostomy was subsequently performed. Colonoscopy revealed mucosal swelling on the anal side a few days after the stool diversion procedure (Fig. 1b). Pathological examination of the colonoscopic biopsy revealed only chronic inflammation. The second abdominal CT examination, performed after 1.5 months, showed circumferential thickening of the pylorus with marked stomach distension (Fig. 1c), and advanced GC was tentatively diagnosed. Esophagogastroduodenoscopy revealed a hyperemic mucosal lesion over the antrum, and the pathology report revealed poorly differentiated GC. Colonoscopy was performed again, and the second pathology report confirmed poorly differentiated GC with colonic submucosal involvement (Fig. 2a). The immunohistochemical analysis results were as follows: CDX-2 (−) (Fig. 2b), CK20 (−) (Fig. 2c), and CK7 (+) (Fig. 2d). The final pathology report stated that the patient had advanced GC with synchronous colonic metastasis. Although the patient received neoadjuvant chemotherapy of 7 cycles of oxaliplatin, folinic acid, and 5-fluorouracil (FOLFOX4) regimen for disease control, he survived for only 6 months.

### Case 2: metachronous colonic metastasis of advanced GC

**Fig. 3:**
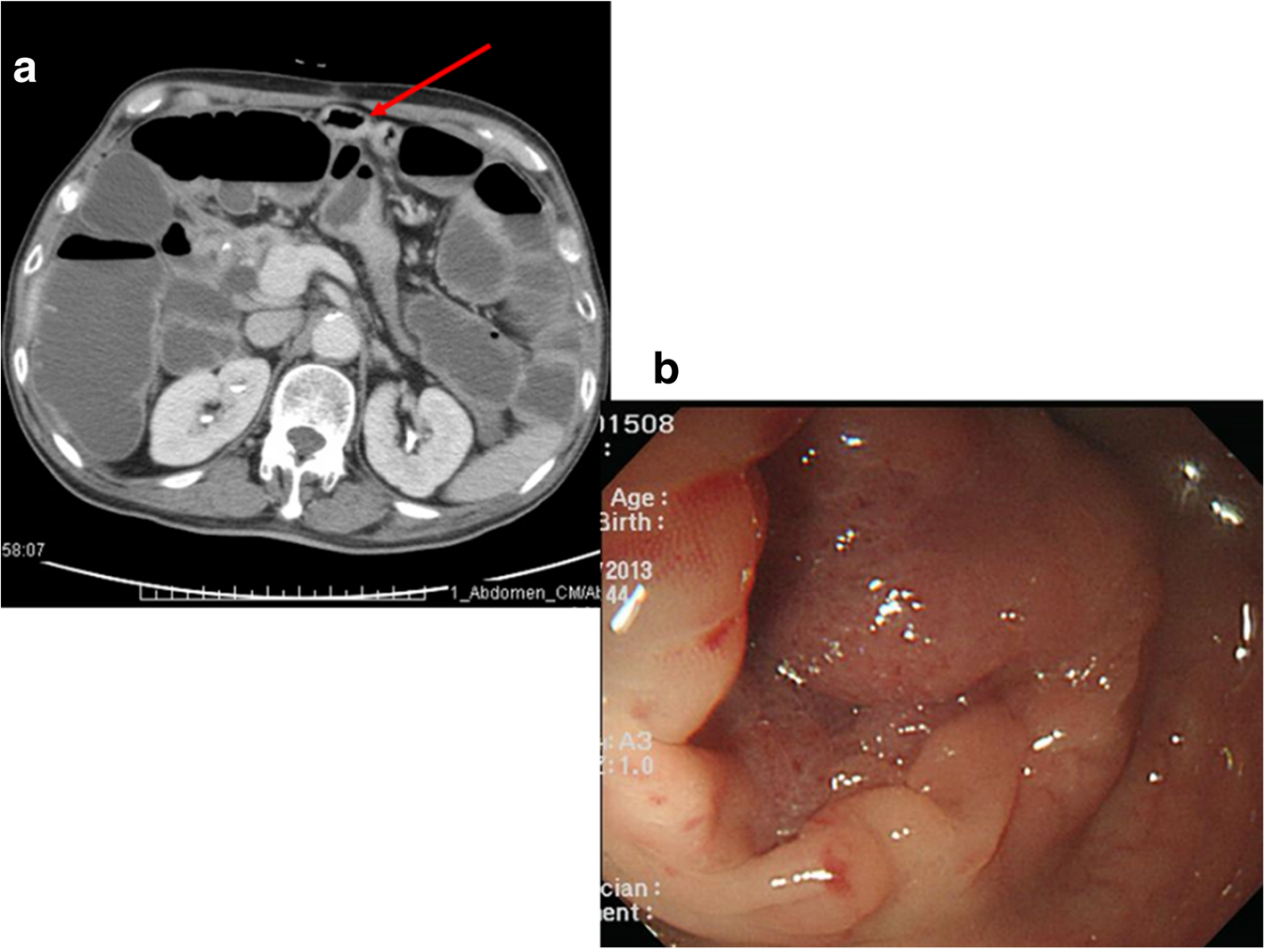
Metachronous colonic metastasis of advanced GC. **a** Abdominal CT showed a circumferential mass lesion with severe distention of the proximal colonic loop and small bowel loop, suggesting colonic metastasis. **b** Colonoscopy revealed mucosal swelling with total obstruction

**Fig. 4:**
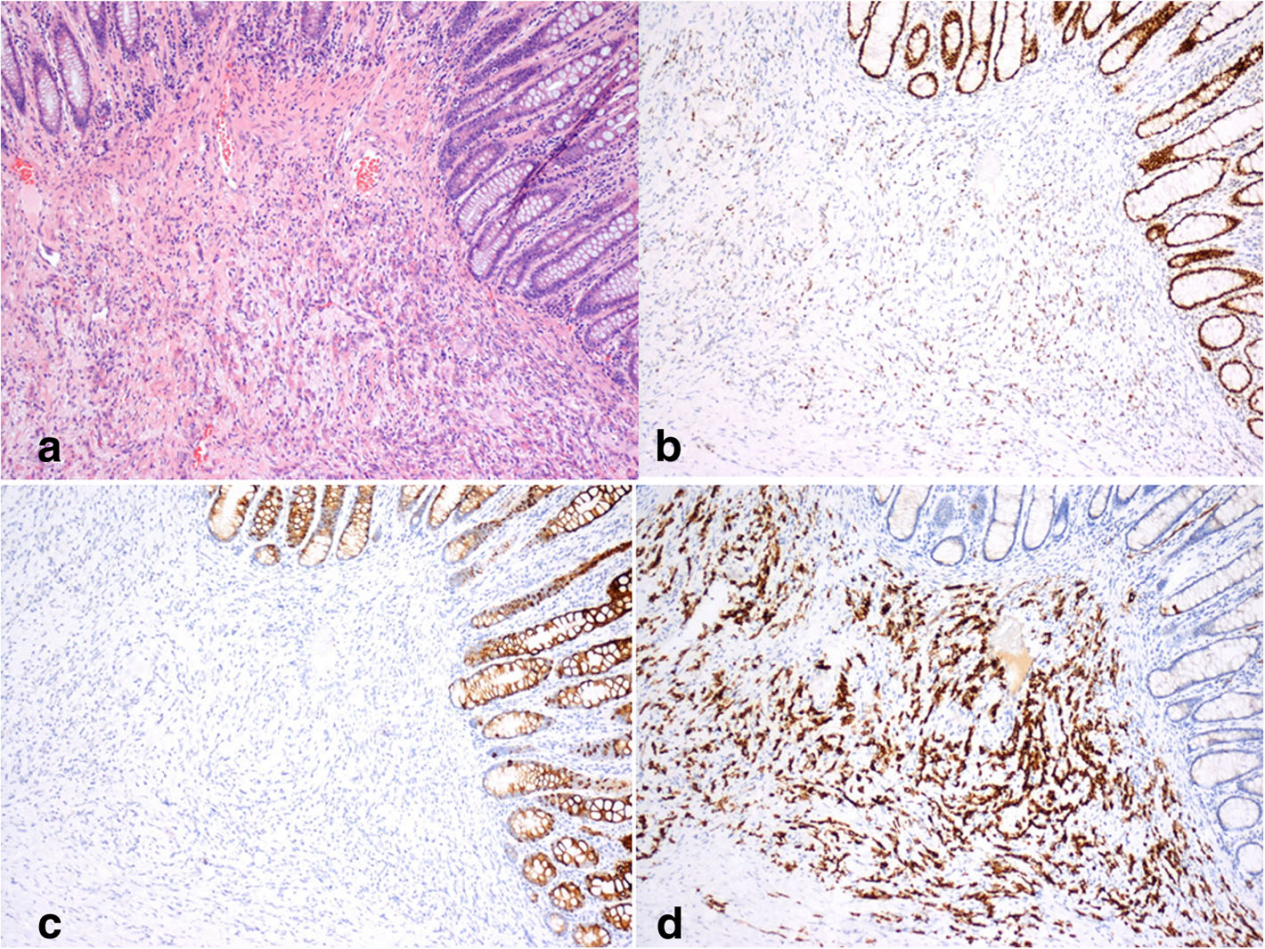
**a** Poorly differentiated carcinoma involving colonic submucosa HE (× 40); **b** CDX-2 staining with weak and faintly positive results (× 100); **c** CK20 staining with negative results (× 100); and **d** CK7 staining with positive results (× 400)

A 78-year-old man had advanced GC with poorly differentiated histology (pT3N3aM0). Eighteen months previously, radical subtotal gastrectomy and Billroth-II gastrojejunostomy had been performed. In addition, he had received neoadjuvant therapy of 12 cycles of the FOLFOX4 regimen. Because the patient had developed progressive abdominal distension, abdominal CT was performed, which showed a circumscribed mass lesion with severe distention at the proximal colonic loop. CT scans also revealed a small bowel loop, which was suspected to be a colon tumor (Fig. 3a). Colonoscopy revealed mucosal swelling characterized by a completely obstructive lesion (Fig. 3b). Transverse colectomy and end-to-end colocolostomy were performed for confirmation. The pathology report confirmed that the patient had metachronous colonic metastasis of advanced GC (Fig. 4a). Immunohistochemical analysis obtained the following marker results: CDX-2 (weak and faintly positive) (Fig. 4b), CK20 (−) (Fig. 4c), and CK7 (+) (Fig. 4d). However, the clinical outcome was negative owing to rapid disease progression. The patient survived for only 6 months after receiving the diagnosis.

## Discussion and conclusions

Colonoscopy revealed that the two patients exhibited different manifestations of colonic masses. We observed only wall thickening and swelling in both cases, with no signs of contact bleeding or centrally ulcerated lesions of the colon. These findings are different from the classic appearance of primary colon cancer upon colonoscopy. Furthermore, intestinal metastasis of GC has rarely been reported [14].

Jang et al. performed a retrospective radiological analysis of 23 GC patients with intestinal metastasis, of whom only 11 were pathologically diagnosed as having colonic metastasis. Most of the patients had poorly differentiated adenocarcinoma or the signet-ring cell type with a propensity to develop into rare intestinal metastasis. Notably, the two patients in the present case report had poorly differentiated adenocarcinoma. Furthermore, intestinal metastasis should be considered by physicians for patients with GC who exhibit wall thickening over segmental bowel and those who exhibit target enhancement and progressive thickening of the enhancing inner layer on CT images [13, 15].

In our cases, segmental bowel wall swelling and progressive thickening of the enhancing inner layer were detected through colonoscopy and CT, respectively. The final diagnosis of colonic metastasis was based on histological features and immunohistochemical analysis. The analyses confirmed the expression of negative CDX-2 in one case, weak and faintly positive CDX-2 in the other, and negative CK20 in both [16, 17]. Moreover, positive CK7 staining was performed to exclude the possibility of prostate cancer.

Cases of GC with colonic metastasis are extremely rare. The survival period for most established cases ranges from 1 to 10 months [4, 9–11]; both patients in our report survived for approximately 6 months after their diagnosis. Furthermore, the prognosis of advanced GC in the two cases was relatively poor even after aggressive treatment. Therefore, colonic metastasis should be considered by physicians if colonoscopy reveals submucosal swelling and segmental bowel wall thickening in patients with advanced GC, particularly in those with poorly differentiated adenocarcinoma or the signet-ring cell type.

## Abbreviations

CT: Computed tomography
FOLFOX4: Oxaliplatin, folinic acid, and 5-fluorouracil
GC: Gastric cancer
